# Soluble α-Klotho as a Novel Biomarker in the Early Stage of Nephropathy in Patients with Type 2 Diabetes

**DOI:** 10.1371/journal.pone.0102984

**Published:** 2014-08-01

**Authors:** Eun Young Lee, Sang Soo Kim, Ji-Sung Lee, In Joo Kim, Sang Heon Song, Seung-Kuy Cha, Kyu-Sang Park, Jeong Suk Kang, Choon Hee Chung

**Affiliations:** 1 Department of Internal Medicine, Soonchunhyang University Cheonan Hospital, Cheonan, Korea; 2 Department of Internal Medicine, Pusan National University Hospital, Busan, Korea; 3 Biostatistical Consulting Unit, Soonchunhyang University Medical Center, Seoul, Korea; 4 Department of Physiology, Yonsei University Wonju College of Medicine, Wonju, Korea; 5 Department of Internal Medicine, Yonsei University Wonju College of Medicine, Wonju, Korea; University of Florida, United States of America

## Abstract

**Objective:**

Although α-klotho is known as an anti-aging, antioxidant, and cardio-renal protective protein, the clinical implications of soluble α-klotho levels in patients with diabetes have not been evaluated. Therefore, this study evaluated whether plasma and urinary α-klotho levels are associated with albuminuria in kidney disease in diabetes.

**Research Design and Methods:**

A total of 147 patients with type 2 diabetes and 25 healthy control subjects were enrolled. The plasma and urine concentrations of α-klotho were analyzed by enzyme-linked immunosorbent assay.

**Results:**

Plasma α-klotho (572.4 pg/mL [95% CI, 541.9–604.6 pg/mL] *vs*. 476.9 pg/mL [95% CI, 416.9–545.5 pg/mL]) and urinary α-klotho levels (59.8 pg/mg creatinine [95% CI, 43.6–82.0 pg/mg creatinine] *vs*. 21.0 pg/mg creatinine [95% CI, 9.7–45.6 pg/mg creatinine]) were significantly higher in diabetic patients than non-diabetic controls. Among diabetic patients, plasma α-klotho concentration was inversely associated with albuminuria stages (normoalbuminuria, 612.6 pg/mL [95% CI, 568.9–659.6 pg/mL], microalbuminuria, 551.8 pg/mL [95% CI, 500.5–608.3 pg/mL], and macroalbuminuria, 505.7 pg/mL [95% CI, 439.7–581.7 pg/mL] (*p* for trend  = 0.0081), while urinary α-klotho levels were remained constantly high with increasing urinary albumin excretion.

**Conclusions:**

Soluble α-klotho levels in plasma and urine may be novel and useful early markers of diabetic renal injury.

## Introduction

Although α-klotho was first described as an anti-aging factor, recent experimental and clinical studies suggest α-klotho also has important pleotroic effects on the kidneys [1]. Soluble α-klotho is derived from the proteolytic cleavage of the extracellular portion of the membrane-bound α-klotho; alternatively, it can be generated directly by the alterative splicing of the α-klotho transcript [2]. It can be measured in blood, urine, and cerebrospinal fluid [1]. Animals with chronic kidney disease have very low renal, plasma, and urinary α-klotho levels [3]. Furthermore, humans with chronic kidney disease exhibit markedly reduced α-klotho in serum [4] and urine [3], [5] in the early stages of kidney disease, progressively decreasing in more advanced stages.

However, with regard to diabetic nephropathy, the role of α-klotho in the pathogenesis of kidney injury has not been fully studied. Renal α-klotho expression is markedly decreased in diabetic nephropathy in humans and mice [6]–[8]. A similar decline is observed in kidney cells treated with methylglyoxal-modified albumin [8]. These findings collectively suggest renal α-klotho deficiency is part of an underlying mechanism involved in diabetic kidney injury. However, the actual role of soluble α-klotho in diabetic kidney disease has not been evaluated.

This study determined whether diabetes influences soluble circulating or urinary α-klotho level and investigated the relationship between these soluble α-klotho levels and albuminuria in patients with type 2 diabetes.

## Research Design And Methods

### Ethics statement

This study was carried out in accordance with the Declaration of Helsinki and study protocol was approved by the Institutional Review Board of Pusan National University Hospital (Busan, Korea). All patients provided their written informed consent before entering the study. Data are available to all interested researchers on request to the Institutional Review Board of Pusan National University Hospital.

### Subjects

A total of 147 consecutive patients with type 2 diabetes were enrolled at outpatient clinics between February 2010 and February 2012. All patients met the following inclusion criteria: age ≥18 years and estimated GFR (eGFR) ≥60 mL min^−1^ 1.73 m^−2^, serum creatinine <1.2 mg/dL, stable renal function status without 2-fold elevation of serum creatinine for at least 5 months, and no history of administration of RAS inhibitors. If patients took RAS inhibitors, these medications were withdrawn and replaced with other antihypertensive agents for at least 2 months before enrollment in this study. A random spot urine sample and a blood sample were obtained from each patient at the clinic visit. Medical histories and anthropometric measurements were also recorded on the same day. The eGFR was calculated using the Modification of Diet in Renal Disease (MDRD) Study formula as follows: MDRD  = 186× (serum creatinine [mg/dL])^−1.154^ × age [years]^−0.203^ [9]; a correction factor of 0.742 was used for women.

Patients with active urinary tract infection; renal disease other than diabetic nephropathy; neoplastic disorders; severe liver dysfunction; active or chronic infection or inflammatory disorders; pregnancy; or a recent (i.e., within 6 months) history of acute myocardial infarction, stroke, or occlusive peripheral vascular disease were excluded. Nondiabetic control subjects were randomly selected from the Center for Health Promotion at Pusan National University Hospital for a comprehensive medical check-up. They were enrolled in this study if they had fasting plasma glucose levels of <100 mg/dL after an overnight fasting, eGFR of ≥60 mL min^−1^ 1.73 m^−2^ and had no prior history of diabetes, renal disease or cardiovascular diseases including hypertension and dyslipidemia.

### Soluble α-klotho enzyme-linked immunosorbent assays

Plasma samples were centrifuged for 15 minutes at 3,000 rpm within 30 minutes of collection; plasma was removed and stored at −70°C until analysis. Urine samples were centrifuged for 10 minutes at 3,000 rpm to remove particulate matter and stored at −70°C until analysis. The plasma and urine concentrations of α-klotho were analyzed by human soluble α-klotho immunoassay kits (Immuno-Biological Laboratories, Gunma, Japan) according to the manufacturer's protocol. All samples were run in duplicate and were within the range of the standard curve (93.75–6,000 pg/mL). Values below the detection limit (6.15 pg/mL) were approximated using the mean value between zero and the lower limit of detection. The intra- and inter-assay coefficients of variation were less than 10%. The levels of urinary α-klotho were expressed as the ratio of urinary α-klotho to urinary creatinine in order to assess the hydration states and renal functions of the patients.

### Statistical analysis

Categorical variables, continuous variables with a normal distribution, and non-normally distributed variables are presented as number (percentage), mean ± SD, and median (interquartile range), respectively. Statistical analyses were performed after log-transforming the data of all skewed variables. Geometric means (i.e., antilogarithms of the transformed means) are presented with 95% CIs. The significance of differences between continuous variables was tested by ANOVA or the Kruskal–Wallis test, with polynomial contrasts for linear trends or Tukey's multiple comparison test where appropriate. Categorical variables were analyzed using the Pearson *χ^2^* test. Pearson correlation analysis was used to determine the correlations between individual variables. Multivariate regression analyses were used to determine the associations of plasma α-klotho with several parameters. All statistical analyses were performed using SPSS version 15.0 (SPSS Inc., Chicago, IL). The level of significance was set at *p*<0.05. All hypothesis tests were 2-sided.

## Results

### Elevated plasma and urinary α-klotho levels in patients with type 2 diabetes

**Figure 1:**
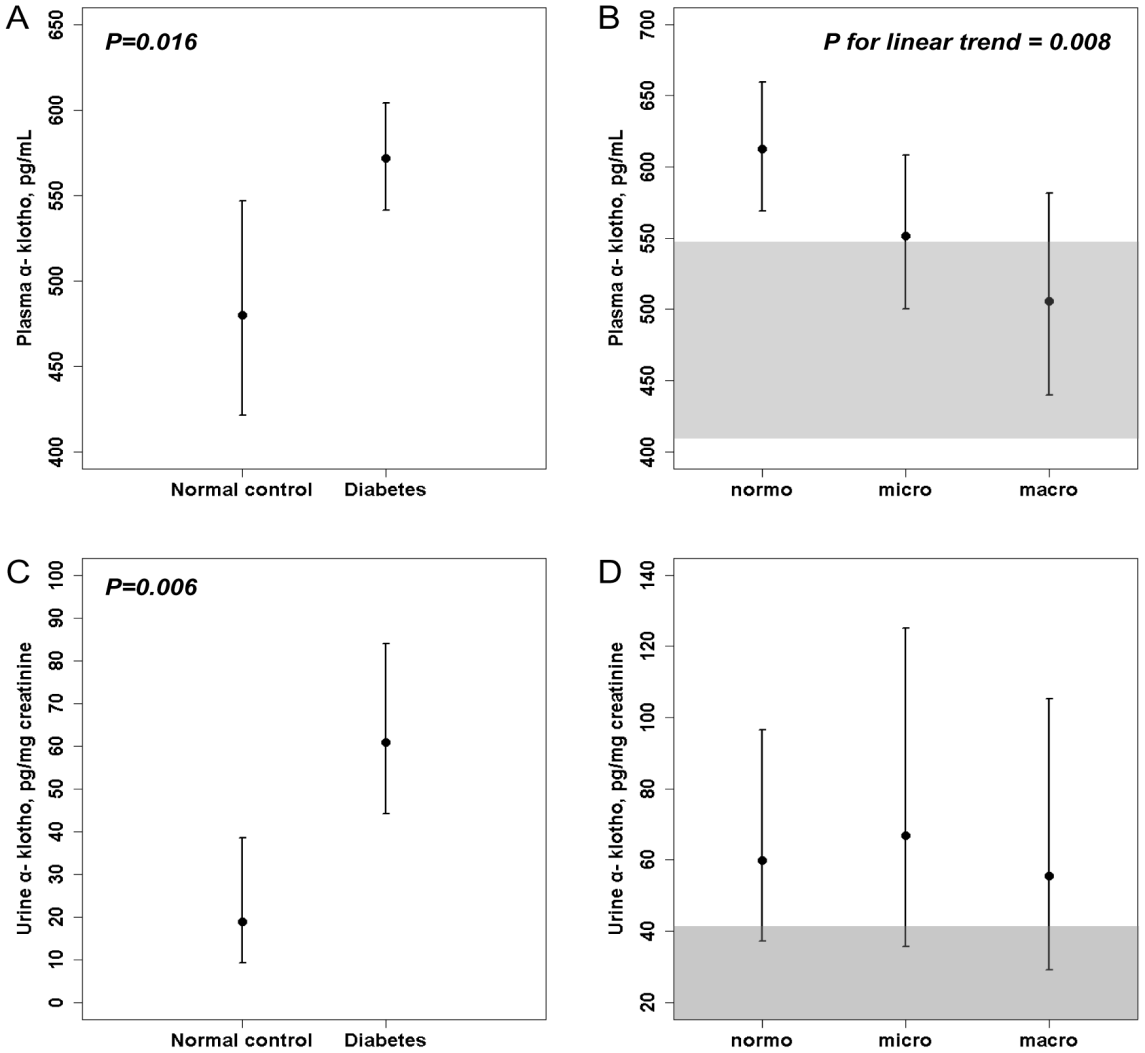
Plasma (A,B) and urine (C,D) levels of soluble α-klotho in normal participants (*n* = 25) and patients with type 2 diabetes (*n* = 147). Plasma and urine α-klotho levels were higher in diabetes patients with relatively preserved renal function than the non-diabetic controls (A,C). The diabetes patients were categorized into 3 groups according to urine ACR: ACR <30 mg/g creatinine (normoalbuminuria group, *n* = 75), ACR 30–299 mg/g creatinine (microalbuminuria group, *n* = 42), and ACR ≥300 mg/g creatinine (macroalbuminuria group, *n* = 30). Plasma α-klotho levels decreased in proportion to urinary albumin excretion, although urinary α-klotho levels were stable with increasing urinary albumin excretion (B,D). Data of non-diabetic control are expressed as a shaded area for the reference (B, D). Data are presented as geometric means and 95% CIs as an error bar plot. *P*-values calculated using the log-transformed values are shown in the graph. normo; normoalbuminuria, micro; microalbuminuria, macro; macroalbuminuria.

A total of 172 subjects were enrolled in this study; their mean age was 55.8±10.4 years (range, 24–82 years), and there were 77 men and 95 women. Age, systolic, diastolic blood pressure, alanine aminotransferase, insulin, and eGFR were significantly higher in diabetic patients than non-diabetic control. Meanwhile, hemoglobin, total bilirubin, and direct bilirubin were significantly lower in diabetic patients than in non-diabetic controls. Other parameters did not differ significantly between the diabetes group and non-diabetic controls (Table 1). Plasma α-klotho was significantly higher in diabetic patients (Fig. 1*A*, 572.4 pg/mL [95% CI, 541.9–604.6 pg/mL] *vs*. 476.9 pg/mL [95% CI, 416.9–545.5 pg/mL], *p* = 0.016). Urinary α-klotho levels were also significantly higher in diabetic patients than in non-diabetic controls (Fig. 1*C*, 59.8 pg/mg creatinine [95% CI, 43.6–82.0 pg/mg creatinine] *vs*. 21.0 pg/mg creatinine [95% CI, 9.7–45.6 pg/mg creatinine], *p* = 0.006).

**Table 1:** Clinical characteristics and laboratory findings of non-diabetic control subjects and type 2 diabetic patients.

	Normal (*n* = 25)	DM (*n* = 147)	*P*-value^†^
Age, years	50.9±7.6	56.6±10.6	0.0101
Sex, male	14(56.0)	63(42.9)	0.2218
BMI, kg/m^2^	23.4±2.9	23.5±3.4	0.8408
SBP, mmHg	119.4±14.3	126.3±14.8	0.0333
DBP, mmHg	74.4±7.9	79.0±10.3	0.0335
Hemoglobin, g/dL	14.7±1.6	13.3±1.6	0.0002
Albumin, g/dL	4.5±0.3	4.4±0.5	0.2321
AST, IU/L	22.2±6.1	23.1±11.5	0.5364
ALT, IU/L	18.4±6.0	24.7±16.9	0.0009
Total cholesterol, mg/dL	193.68±33.60	180.03±40.64	0.1141
HDL cholesterol, mg/dL	55.32±13.42	50.04±16.87	0.1392
Triglycerides, mg/dL*	110.35(87.61 138.99)	140.78(126.98 156.09)	0.0726
Total bilirubin, mg/dL	1.07±0.36	0.64±0.21	<.0001
Direct bilirubin, mg/dL	0.21±0.07	0.14±0.05	0.0002
Uric acid, mg/dL	5.10±0.91	4.75±1.31	0.1027
Calcium, mg/dL	9.44±0.45	9.36±0.49	0.4258
Phosphorus, mg/dL	3.50±0.63	3.65±0.60	0.2621
Serum creatinine, mg/dL	0.85±0.14	0.80±0.17	0.1885
eGFR, mL/min/1.73 m^2^*	85.49(81.70 89.45)	90.62(87.4293.94)	0.0449
C-reactive protein, mg/L*	0.05 (0.04 0.08)	0.08 (0.06 0.09)	0.1924
Insulin, µIU/mL	1.24(0.63 2.23)	5.83(3.35 11.62)	<.0001

Values are mean ± SD, number of patients (%), median (interquartile range), and geometric means (95% CI) unless otherwise indicated. DM, diabetes mellitus; SBP, systolic blood pressure; DBP, diastolic blood pressure; AST, aspartate aminotransferase; ALT, alanine aminotransferase; eGFR, estimated glomerular filtration rate. *P-values were calculated using log-transformed values. ^†^ *P*-values were calculated by Student's *t*-test, Mann-Whitney u-test or Pearson *χ^2^* test where appropriate.

### Inverse correlation between plasma α-klotho levels and albuminuria in patients with type 2 diabetes

Categories of progressively worse albuminuria were linearly associated with lower plasma α-klotho levels. The diabetic patients were categorized into 3 groups according to urine albumin creatinine ratio (ACR): ACR <30 mg/g creatinine (normoalbuminuria group, *n* = 75), ACR 30–299 mg/g creatinine (microalbuminuria group, *n* = 42), and ACR ≥300 mg/g creatinine (macroalbuminuria group, *n* = 30) (Table 2). Age, sex, BMI, diastolic blood pressure, aspartate aminotransferase, alanine aminotransferase, HDL cholesterol, uric acid, phosphorous, serum creatinine, eGFR, C-reactive protein, and insulin did not differ significantly among the 3 groups.

**Table 2:** Baseline clinical and laboratory parameters in patients with type 2 diabetes according to albuminuria status.

	Normoalbuminuria (*n* = 75)	Microalbuminuria (*n* = 42)	Macroalbuminuria (*n* = 30)	*P*-value^†^
Age, years	56.8±10.5	56.9±11.9	55.8±9.1	0.8995
Sex, male	31(41.3)	15(35.7)	17 (56.7)	0.1938
Duration of diabetes, years	4^ab^(2–6)	8^a^(5–14)	9^b^(6–15)	0.0007
Diabetic retinopathy, %	17(22.7)	19(45.2)	17(56.7)	0.0016
Diabetic neuropathy, %	8(10.7)	20(47.6)	13(43.3)	<.0001
BMI, kg/m^2^	23.4±3.0	23.7±4.2	23.4±3.4	0.8957
SBP, mmHg	123.5^a^±12.0	126.1±12.3	133.4^a^±21.2	0.0077
DBP, mmHg	78.0±8.8	78.3±10.5	82.5±12.6	0.1073
HbA_1c_, %	7.8^ab^±1.4	8.4^a^±1.6	8.6^b^±2.0	<.0001
Glycated albumin, %*	18.40^ab^(17.24 19.64)	24.32^a^(22.15 26.70)	21.87^b^(18.68 25.61)	<.0001
Hemoglobin, g/dL	13.6^a^±1.3	13.3±1.6	12.7^a^±2.2	0.0297
Albumin, g/dL	4.5^a^±0.3	4.4^b^±0.4	4.1^ab^±0.7	<.0001
AST, IU/L	23.3±8.7	23.0±14.3	22.9±13.2	0.9882
ALT, IU/L	24.7±13.6	25.2±21.2	23.8±18.5	0.9417
Total cholesterol, mg/dL	170.44^a^±37.18	181.57±37.83	201.87^a^±45.03	0.0013
LDL cholesterol, mg/dL	98.6^a^±30.6	111.2±34.4	123.8^a^±57.7	0.0090
HDL cholesterol, mg/dL	49.0±15.7	49.9±12.9	52.8±23.7	0.5944
Triglycerides, mg/dL*	128.11^a^(110.14 149.01)	139.63(117.92 165.33)	180.31^a^(141.72 229.41)	0.0428
Total bilirubin, mg/dL	0.66^a^±0.21	0.67^b^±0.19	0.53^ab^±0.23	0.0067
Direct bilirubin, mg/dL	0.15^a^±0.05	0.15^b^±0.05	0.12^ab^±0.06	0.0076
Uric acid, mg/dL	4.6±1.2	4.7±1.4	5.2±1.3	0.1393
Calcium, mg/dL	9.39±0.37	9.44^a^±0.46	9.15^a^±0.72	0.0362
Phosphorus, mg/dL	3.56±0.57	3.73±0.60	3.74±0.67	0.2406
Serum creatinine, mg/dL	0.79±0.15	0.77±0.16	0.85±0.20	0.1387
eGFR, mL/min/1.73 m^2^*	90.3(86.5 94.2)	92.3(85.7 99.5)	89.2(80.4 98.8)	0.7856
C-reactive protein, mg/L*	0.07(0.05 0.09)	0.09(0.06 0.13)	0.07(0.04 0.12)	0.5280
Insulin, µIU/mL	5.87(3.35 11.62)	5.19(1.49 13.05)	7.54(4.26 11.70)	0.5165
Urine ACR, mg/g*	9.85^ab^(6.74 9.85)	103.04^ac^ (68.11 103.04)	1131.12^bc^(609.53 1131.12)	<.0001
Urine PCR, mg/g*	100.67^ab^(83.22 100.67)	356.16^ac^(222.33 356.16)	2261.86^bc^(1146.68 2261.86)	<.0001

Values are mean ± SD, number of patients (%), median (interquartile range), and geometric means (95% CI) unless otherwise indicated. DR, diabetic retinopathy; DN, diabetic neuropathy; SBP, systolic blood pressure; DBP, diastolic blood pressure; TC, total cholesterol; TG, triglycerides; TB, total bilirubin; DB, direct bilirubin; UA, uric acid; Ca, calcium; P, phosphorous; Scr, serum creatinine; eGFR, estimated glomerular filtration rate; CRP, C-reactive protein; ACR, Urine albumin creatinine ratio; PCR, Urine protein creatinine ratio. *P-values were calculated using log-transformed values. ^†^ *P*-values were calculated by ANOVA or Pearson *χ^2^* test where appropriate. The same superscripted letters indicate statistical significance according to Tukey's multiple comparison test.

The mean plasma α-klotho concentration in the normoalbuminuria group was 612.58 pg/mL compared to 505.70 pg/mL in the macroalbuminuria group (average difference, 106.88 pg/mL). Fig. 1 shows a clear inverse association between plasma α-klotho level and albuminuria level. Plasma α-klotho concentrations were the highest in the normoalbuminuria group and tended to decrease with increasing degrees of albuminuria (Fig. 1*B*, *p* for linear trend  = 0.008). In contrast to plasma α-klotho levels, no albuminuria level was significantly associated with urinary α-klotho creatinine ratio (Fig. 1*D*).

### Correlations between plasma α-klotho levels and other parameters in patients with type 2 diabetes

As shown in Table 3, plasma α-klotho was significantly correlated with hemoglobin, aspartate aminotransferase, alanine aminotransferase, and insulin. Meanwhile, plasma α-klotho was negatively correlated with phosphorus, urine protein creatinine ratio, and urine ACR (*P<*0.05). However, there were no significant correlations between plasma α-klotho and HbA_1C_, eGFR, or urine α-klotho. We performed multivariate regression analyses for the associations of plasma α-klotho with several parameters. In a multiple linear regression analysis, plasma α-klotho was significantly associated with hemoglobin (*r* = 0.01344, *P* = 0.0095) and urine ACR (*r* = 0.00154, *P*<0.0001, Table 4).

**Table 3:** Correlations between plasma α-klotho levels and other parameters in patients with diabetes (n = 147).

	Plasma klotho*	
	*r*	*P*-value
BMI, kg/m^2^	0.098	0.2398
SBP, mmHg	−0.013	0.8740
DBP, mmHg	0.046	0.5820
HbA_1c_, %	0.046	0.5773
Glycated albumin, %*	0.069	0.4076
Hemoglobin, g/dL	0.287	0.0005
Albumin, g/dL	0.050	0.5511
AST, IU/L	0.243	0.0030
ALT, IU/L	0.194	0.0188
Total cholesterol, mg/dL	0.005	0.9547
LDL cholesterol, mg/dL	0.040	0.6308
HDL cholesterol, mg/dL	−0.013	0.8763
Triglycerides, mg/dL*	−0.116	0.1622
Total bilirubin, mg/dL	0.137	0.0978
Direct bilirubin, mg/dL	0.155	0.0626
Uric acid, mg/dL	−0.117	0.1604
Calcium, mg/dL	0.060	0.4671
Phosphorus, mg/dL	−0.240	0.0037
Serum creatinine, mg/dL	0.059	0.4749
eGFR, mL/min/1.73 m^2^*	−0.018	0.8265
C-reactive protein, mg/L*	−0.080	0.3335
Insulin, µIU/mL	0.183	0.0273
Urine ACR, mg/g*	−0.214	0.0093
Urine PCR, mg/g*	−0.237	0.0040
Urine klotho, pg/mg*	−0.011	0.8967

SBP, systolic blood pressure; DBP, diastolic blood pressure; AST, aspartate aminotransferase; ALT, alanine aminotransferase; eGFR, estimated glomerular filtration rate; ACR, albumin creatinine ratio; PCR, protein creatinine ratio. *Log transformed data before analysis, *r:* Pearson correlation coefficient.

**Table 4:** Multiple linear regression analysis between plasma α-klotho levels and other parameters in patients with diabetes (*n* = 147).

	Plasma Klotho*		
	β	SE	P-value
Age, years	0.00038	0.00066	0.5690
Hemoglobin, g/dL	0.01344	0.00511	0.0095
ALT, IU/L	−0.00035	0.00042	0.4047
Phosphorus, mg/dL	0.02243	0.01211	0.0661
Total bilirubin, mg/dL	−0.02343	0.03553	0.5108
Insulin, µIU/mL	−0.00009	0.00014	0.5321
Urine ACR, mg/g	0.00154	0.00003	<.0001
Urine PCR, mg/g	0.00000	0.00001	0.9961

ALT, alanine aminotransferase; ACR, albumin creatinine ratio; PCR, protein creatinine ratio. *Log transformed data before analysis.

## Conclusions

This study is the first to demonstrate the plasma and urine levels of soluble α-klotho are significantly elevated in the diabetic patients with relatively preserved renal function compared to control subjects. The results also show plasma α-klotho levels decreased in proportion to urinary albumin excretion, although urinary α-klotho levels were stable with increasing urinary albumin excretion.

α-Klotho is a single-pass transmembrane protein that is highly expressed in the kidneys and is known to act as a co-receptor for fibroblast growth factor-23 [1]. Circulating soluble α-klotho can be generated directly by the alterative splicing of the α-klotho transcript or the extracellular domain of membrane α-klotho can be released from membrane-anchored α-klotho on the cell surface [2]. Unlike membrane α-klotho, which functions as a co-receptor for fibroblast growth factor-23, soluble α-klotho acts as a hormonal factor and plays important roles in anti-aging, anti-oxidation, ion transport modulation, and Wnt signaling. Previous studies aiming to clarify the role of α-klotho as a potential biomarker of kidney injury show the blood and urinary concentrations of α-klotho decrease early in the course of chronic kidney disease in mice with experimentally induced chronic kidney disease [3] as well as humans [4]. Although blood α-klotho concentration was found to be linearly associated with eGFR in previous studies [3], [4], plasma α-klotho was not associated with the eGFR in the present study. The reason for this discrepancy is that only patients with an eGFR >60 mL min^−1^ 1.73 m^−2^ were enrolled; the geometric mean of eGFR was 90.6 mL min^−1^ 1.73 m^−2^ in the present study. The concentration of α-klotho in human urine is estimated to be 20–200 pM [10]. Urinary α-klotho is known to be correlated with eGFR and is reported to be a surrogate marker of functioning nephrons in the patients with chronic kidney disease [3], [5]. The present finding that urinary α-klotho was higher in the diabetic patients whose eGFR is also higher than that of normal people, further corroborates this.

Meanwhile, little is known about circulating α-klotho levels in diabetes-related nephropathy. Recent studies in patients with diabetes report conflicting data. One study found serum α-klotho level was not significantly different between patients with diabetes without nephropathy and non-diabetic controls [11], [12]. In contrast, another study reports a significant reduction in serum klotho levels in patients with glycated hemoglobin (HbA1c) levels ≥6.5% compared to control samples (HbA1c <6.5%) [13].

Kacso *et al.* [12] report α-klotho decreases in early chronic kidney disease and increases thereafter in the diabetic patient. However, they did not evaluate the association between soluble α-klotho levels and the extent of albuminuria in the early stage of diabetic nephropathy, specifically in patients with normal renal function. Asai *et al.* previously showed that renal α-klotho levels were significantly decreased in early diabetic nephropathy patients [7], however, they've never compared renal α-klotho levels between diabetic patients and normal control. They've just showed reduction in renal α-klotho levels in diabetic nephropathy patients than patients with minimal change disease or IgA nephropathy. Furthermore, the mean age of diabetic nephropathy patients was significantly older than patients with minimal change disease and IgA nephropathy. They also showed that renal α-klotho levels were significantly decreased in diabetic mice at 8 weeks after development of diabetes mellitus. They showed that albuminuria was increased at 2, 4, 6, and 8 weeks after onset of diabetes, however, renal α-klotho levels were not decreased until 4 weeks after development of diabetes. And they've not reported the renal α-klotho levels in early stage of albuminuric diabetic mice before 4 weeks of diabetes.

Zhao *et al.* showed decreased renal klotho expression in *db/db* mouse [8]. Although they did not indicate the levels of albuminuria or renal function data, they used *db/db* mouse at 20 weeks of age, which is regarded as relatively late stage of diabetic nephropathy. Deveraj *et al*. reported that soluble fraction of klotho was decreased in diabetic patients than non-diabetic controls, however they never mentioned the albuminuria status of their diabetic patients [13]. According to our data, there was a clear inverse association between plasma α-klotho level and albuminuria level in diabetic patients with relatively preserved renal function. van Ark J *et al*. measured circulating α-klotho levels in diabetes patients. Although they reported that circulating α-klotho levels were not changed in diabetic patients compared to control, their sample size was very small (*n* = 35) and they never mentioned the albuminuria status of their diabetic patients [11]. In the present study, plasma α-klotho levels in patients with type 2 diabetes were highest in the normoalbuminuria stage and decreased with increasing urinary albumin excretion. It is surprising that the plasma α-klotho levels in the macroalbuminuria group were still comparable with those in the non-diabetic controls.

Both acute kidney injury and chronic kidney disease exhibit renal and systemic α-klotho deficiency. Levels of α-klotho plummet very early and severely in acute kidney injury, representing a pathogenic factor that exacerbates acute kidney damage [14]. In chronic kidney disease, α-klotho deficiency significantly impacts the progression of renal disease as well as extrarenal complications [3], [4]. Meanwhile, soluble α-klotho levels in plasma and/or urine may serve as early biomarkers of kidney parenchymal injury [15].

Recent studies indicate the potential contribution of absolute α-klotho deficiency to acute and chronic kidney injury [14], [15]. Emerging evidence suggests α-klotho deficiency is an early biomarker of kidney disease as well as a pathogenic factor. α-Klotho deficiency is associated with progression and chronic complications in chronic kidney disease, including vascular calcification, cardiac hypertrophy, and secondary hyperparathyroidism; in particular, α-klotho deficiency induces resistance to fibroblast growth factor-23 and predisposition to hyperphosphatemia, which represents a critical feature of chronic kidney disease [1]. In the present study, plasma α-klotho concentrations tended to decrease with increasing degrees of albuminuria. Surprisingly, plasma α-klotho levels in diabetes-related nephropathy were still not lower than those in the normal controls, whereas they were lower in chronic kidney disease patients than normal controls in previous studies. Although absolute plasma α-klotho levels were not lower than normal, it is possible these levels are insufficient to prevent albuminuria in the microalbuminuria and macroalbuminuria stages of diabetes-related kidney disease. The results of the present study may help further elucidate the role of α-klotho in the development and progression of albuminuria in type 2 diabetes.

It is worth noting that the significance of urine α-klotho concentration as an early biomarker has not been evaluated in chronic kidney disease. The most important finding of the present study is that for the first time, high urine α-klotho levels were found to be associated with diabetes in humans even in the normoalbuminuria stage.

Nevertheless, the underlying mechanisms explaining the present results require further investigation. The present results may be explained by increased α-klotho synthesis or its cleavage process, although requires further study. The extracellular domain of α-klotho protein is subject to ectodomain shedding and is released into the blood and urine [1]; therefore, it may function as a hormone [16]. Hyperglycemia does not affect renal α-klotho production per se, because high glucose does not alter α-klotho expression in kidney cells and diabetes does not affect renal α-klotho mRNA expression in mice [11]. The lack of an association between HbA_1c_ or glycated albumin with soluble α-klotho concentrations in the present study also corroborates previous observations. Insulin can increase soluble α-klotho concentration through the cleavage and release of the extracellular domain of α-klotho [17]. In type 2 diabetes at early stage, soluble α-klotho level is increased in plasma that may result in increased amount of α-klotho protein in urine. α-Klotho protein is expressed in both apical and basolateral membrane of kidney tubule [18]. Soluble α-klotho level may be determined by two possible mechanisms; 1) cleavage of α-klotho protein by proteases such as ADAM10 or 17 [17] and 2) secretion of splice variant form of α-klotho into blood or urine. Extracellular domain of α-klotho can be released into urine and blood from apical and basolateral membranes, respectively. Insulin receptor is also expressed in both apical and basolateral membrane of kidney tubular cells. At early stagy of type 2 diabetes, blood insulin level is increased that can stimulate the cleavage and release of the extracellular domain of α-klotho [17] into blood and/or urine. In this study, blood insulin level was also increased in diabetic patients than non-diabetic controls as expected. Increased level of soluble α-klotho may be filtered in glomeruli and present in urine. Cha *et al.* [10] reported that intraperitoneal administration of soluble klotho increased urinary K^+^ excretion in rat by ROMK channel activation which is expressed in apical membrane. Interestingly, epitope-tagged klotho was appeared in urine at 2 hr after intravenous administration [19] indicating that klotho protein may be filtered in glomeruli and regulates ROMK channel from luminal side.

We found an interesting decrease in hemoglobin concentration from 13.6 g/dL in patients with normoalbuminuria to 13.3 g/dL in patients with microalbuminuria and then low to 12.7 g/dL in macroalbuminuria (Table 2). The decrease of hemoglobin concentration in the absence of significant excretory dysfunction has also been demonstrated in other study [20]. The decrease in hemoglobin in macroalbuminuria as compared to normoalbuminuria cannot be explained by reduced renal function as there was no significant difference in eGFR between the groups (Table 2). Our and the previous findings may suggest that erythropoietin deficiency, which has a major etiological role in the anemia associated with renal failure, begins even before there is evidence of deterioration in renal function. Other possibility includes eryptosis. Enhanced eryptosis, suicidal erythrocyte death, is observed in diabetes [21] and eryptosis is further enhanced in klotho-deficient mice [22].

Plasma and urinary α-klotho were not correlated in a previous study [5] or the present study.

Exogenous supplementation or stimulation of endogenous α-klotho may prevent and/or ameliorate kidney injury and mitigate chronic kidney disease development. The correction of α-klotho deficiency may delay the progression and forestall the development of extrarenal complications in chronic kidney disease. Angiotensin II receptor blocker treatment was recently shown to increase blood α-klotho levels while reducing albuminuria in type 2 diabetes with nephropathy [23], [24]. The findings that both exogenous soluble α-klotho administration and overexpression of membranous α-klotho in kidney cell culture suppress NF-κB activation and subsequent inflammatory cytokine production in the response to TNF-α stimulation suggest α-klotho serves as an anti-inflammatory modulator [8]. Therefore, preventing deceases in α-klotho and α-klotho supplementation are potential novel therapeutic strategies for early diabetic nephropathy. In multiple experimental models of chronic kidney disease, the replacement or endogenous upregulation of α-klotho protects the kidneys from renal insults, preserves kidney function, and suppresses renal fibrosis. Thus, α-klotho is a highly promising candidate early biomarker as well as a novel therapeutic agent for chronic kidney disease [2].

The results of this study are subject to some limitations. First, the sample size was relatively small. We measured the urinary levels of α-klotho with single random spot urine samples, although urine samples were collected at the outpatient clinic from patients without illness or renal diseases besides diabetic nephropathy; moreover, a moderate linear association was observed between the amount of urine α-klotho in 24 hours and urinary α-klotho/creatinine ratio in random urine specimens (*r* = 0.726, *p*<0.01) [5]. Despite these limitations, it is noteworthy that blood and urine α-klotho concentrations can easily be checked and used to assess the development of diabetic nephropathy prior to the onset of microalbuminuria, which is the earliest sign of diabetic nephropathy in clinical settings. Second, In addition, we measured and evaluated plasma and urinary α-klotho concentrations at a single time point in this cross-sectional study. Therefore, it is unclear whether plasma and/or urine α-klotho causes albuminuria in diabetes. Furthermore, all enrolled patients on medication with RAS inhibitors had a sufficient washout period for these drugs in order to role out the effect of RAS inhibitors on plasma and/or urinary klotho levels and albuminuria.

In conclusion, the results of the present study suggest plasma and urinary α-klotho may be the early markers for predicting renal injury in patients with type 2 diabetes and we need to do long-term prospective study in order to elucidate the role of α-klotho in the pathophysiological mechanisms of the development and progression of albuminuria in type 2 diabetes.
